# Neurites containing the neurofilament-triplet proteins are selectively vulnerable to cytoskeletal pathology in Alzheimer’s disease and transgenic mouse models

**DOI:** 10.3389/fnana.2013.00030

**Published:** 2013-09-26

**Authors:** Stanislaw Mitew, Matthew T. K. Kirkcaldie, Tracey C. Dickson, James C. Vickers

**Affiliations:** ^1^Wicking Dementia Research and Education Centre, University of TasmaniaHobart, TAS, Australia; ^2^School of Medicine, University of TasmaniaHobart, TAS, Australia; ^3^Menzies Research Institute, University of TasmaniaHobart, TAS, Australia

**Keywords:** neurofilament triplet, interneuron, dystrophic neurites, amyloid plaque, Alzheimer’s disease

## Abstract

Amyloid-β plaque accumulation in Alzheimer’s disease (AD) is associated with dystrophic neurite (DN) formation and synapse loss in principal neurons, but interneuron pathology is less clearly characterized. We compared the responses of neuronal processes immunoreactive for either neurofilament triplet (NF^+^) or calretinin (CR^+^) to fibrillar amyloid (Aβ) plaques in human end-stage and preclinical AD, as well as in APP/PS1 and Tg2576 transgenic mouse AD models. Neurites traversing the Aβ plaque core, edge, or periphery, defined as 50, 100, and 150% of the plaque diameter, respectively, in human AD and transgenic mouse tissue were compared to age-matched human and wild-type mouse controls. The proportion of NF^+^ neurites exhibiting dystrophic morphology (DN) was significantly larger than the proportion of dystrophic CR^+^ neurites in both human AD and transgenic mice (*p* < 0.01). Additionally, the number of NF^+^, but not CR^+^, DNs, correlated with Aβ plaque size. We conclude that CR^+^ interneurons appear to be more resistant than NF^+^ neurons to AD-mediated cytoskeletal pathology.

## INTRODUCTION

Alzheimer’s disease (AD) is commonly associated with a cascade of neuronal cytoskeletal alterations – neurofibrillary tangles (NFTs), neuropil threads and dystrophic neurite (DN) formation, causing spine and synapse loss – as well as overt neuronal degeneration (Spires et al., 2005; Adalbert et al., 2009; Vickers et al., 2009). These pathological changes develop in a characteristic spatiotemporal progression across the cortex in most human cases (Braak et al., 2011), and to some extent in AD mouse models (Blanchard et al., 2003), suggesting a differential subregional and cellular susceptibility to AD. DNs are intimately associated with extracellular depositions of amyloid β (Aβ), known as plaques (Dickson et al., 1999; Woodhouse et al., 2009b) and present as swollen tortuous neurites 10–60 μm in diameter, with variable morphology and composition depending on the pathological stage of AD (Vickers et al., 1996; Su et al., 1998; Woodhouse et al., 2009b). In preclinical AD cases and transgenic mice, Aβ plaque-associated DNs are predominantly labeled with antibodies to neurofilament triplet proteins (NFs) and α-internexin, whereas in end-stage AD, subgroups of DNs contain NFs, abnormal tau protein, or a rim of NFs around a tau core (Benzing et al., 1993; Su et al., 1996; Dickson et al., 1999, 2005). This suggests that DN composition may shift from NF/α-internexin to predominantly tau, as disease progresses (Blanchard et al., 2003; Vickers et al., 2009). This progression may imply similar shifts elsewhere in affected neurons.

Neurofilament triplet proteins belong to the type IV intermediate filament protein family, and, in the neocortex of many mammalian species, are predominantly expressed by a pyramidal subpopulation in layers 2–6 making corticocortical connections (Vickers and Costa, 1992; Hof et al., 1995; Van der Gucht et al., 2007; Paulussen et al., 2011). The NF “triplet” refers to three genetically and structurally interrelated subunits [68 kDa (NF-L), 160 kDa (NF-M), and 200 kDa (NF-H)] that are typically co-expressed, and which co-polymerize to form intermediate filaments (Szaro and Strong, 2010). In the rat neocortex, NF-immunopositive (NF^+^) neurons account for approximately 10–13% of all neurons (Kirkcaldie et al., 2002), whereas 20–30% of human temporal cortex neurons are NF^+^ (Hof et al., 1990).

The effect of AD on NF^+^ neuronal populations has been widely studied as a possible insight to the basis of neuronal vulnerability, and perhaps the pathological mechanisms of AD. SMI32, an antibody which recognizes dephosphorylated epitopes of the NF-M and NF-H subunits but does not cross-react with tau, labels a subset of layer 2, 3, and 5 pyramidal neurons which may be particularly susceptible to neurofibrillary pathology (Lewis et al., 1987; Morrison et al., 1987; Hof and Morrison, 1990; Hof et al., 1990, 1995; Mann, 1996). NF^+^ neurons in superior frontal and inferior medial temporal association cortices (Hof et al., 1990), primary and secondary visual cortex (Hof and Morrison, 1990), and hippocampal and entorhinal regions (Vickers et al., 1992, 1994) show a high degree of vulnerability to NFT formation and degeneration. Interestingly, cortical neurons that lack the NF-triplet, including most inhibitory interneurons, do not develop NFTs and show a much lower susceptibility to degeneration in AD (Hof et al., 1991, 1993; Fonseca and Soriano, 1995; Sampson et al., 1997). Conversely, those subpopulations of inhibitory neurons which do express the NF-triplet are more likely to develop NFTs in AD (Hof et al., 1993; Sampson et al., 1997; Leuba et al., 1998).

To further examine if NF^+^ neurons and their processes are selectively vulnerable to AD pathology, we performed a morphological analysis of neurites in early and end-stage AD cases, as well as in two widely used transgenic mouse models, APP/PS1 and Tg2576, that develop AD-like Aβ plaque pathology. We also analyzed calretinin-immunopositive (CR^+^) neurites as a non-neurofilament comparison: although a small population of Cajal–Retzius cells in layer 1 are both CR^+^ and NF^+^ (Fonseca and Soriano, 1995), CR^+^ interneurons in the layers we studied are from NF^-^ neurons (del Rio and DeFelipe, 1997; Sampson et al., 1997).

## MATERIALS AND METHODS

### HUMAN TISSUE

Human brain tissue was acquired from the Sun Health Research Institute (AZ, USA) and the National Health and Medical Research Council Brain Bank (Adelaide, Australia), meeting all necessary ethical approvals as previously described (Dickson et al., 1999; Woodhouse et al., 2009b). Blocks of inferior temporal cortex, a major site of Aβ deposition (Braak et al., 2011), were immersion-fixed with either 10% formalin or 4% paraformaldehyde. Human tissue comprised six sporadic AD cases meeting CERAD diagnostic criteria (“end-stage” AD; Braak stage IV–V), six non-demented cases with neocortical plaque pathology but no NFTs (i.e., Braak stage III), termed “preclinical” AD (Price et al., 2009), and five age-matched non-demented cases lacking cortical Aβ plaques or neurofibrillary pathology (**Table 1**).

**Table 1:** Human brain cases utilized for immunohistochemistry and analysis.

Type	Age	Gender	Postmortem interval (h)	Cortical region	Cause of death	Source^*^	Plaque load (%)
Sporadic AD	92	F	2.25	ITG	Pneumonia	SHRI	3.59
Sporadic AD	74	F	2.0	ITG	Pneumonia	SHRI	3.58
Sporadic AD	74	M	2.75	ITG	Respiratory arrest	SHRI	2.81
Sporadic AD	83	M	2.75	ITG	AD	SHRI	2.94
Sporadic AD	66	M	2.75	ITG	AD	SHRI	1.96
Sporadic AD	84	F	3.0	ITG	AD	SHRI	2.98
Preclinical AD	90	M	2.25	ITG	Respiratory arrest	SHRI	2.14
Preclinical AD	81	F	3.0	ITG	Cardiac arrest	SHRI	2.73
Preclinical AD	84	M	3.0	ITG	Cardiopulmonary arrest	SHRI	3.03
Preclinical AD	78	M	2.25	ITG	Postoperative	SHRI	1.33
Preclinical AD	91	M	3.0	ITG	Cardiac arrest	SHRI	1.42
Preclinical AD	74	M	31.5	ITG	Cardiac arrest	NTRC	2.13
Control	58	M	27.0	TG	Cardiac arrest	U Syd	–
Control	51	M	23.5	TG	Pulmonary embolus	U Syd	–
Control	47	M	27.5	TG	Pneumonia	U Syd	–
Control	65	M	16.0	TG	Cardiopulmonary arrest	U Syd	–
Control	71	M	32.5	TG	Postoperative	U Syd	–

* SHRI, Sun Health Research Institute (AZ, USA); NTRC, National Tissue Resource Centre (Melbourne, Australia); U Syd, Department of Pathology, University of Sydney (Australia).

### MOUSE TISSUE

All mouse procedures were approved by the Animal Ethics Committee of the University of Tasmania and are in accordance with the Australian Code of Practice for the Care and Use of Animals for Scientific Purposes. Briefly, 12-month-old Tg2576 (APP_Swe670/671_; Hsiao et al., 1996), APP/PS1 (APP_Swe_,PSEN1dE9; Jankowsky et al., 2004), and age-matched C57BL/6 wild-type mice (*n* = 5, for each group) were terminally anesthetized (sodium pentobarbitone, 140 mg/kg, i.p.), transcardially perfused (0.01 M phosphate-buffered saline, PBS, then 4% paraformaldehyde in PBS). Brains were post-fixed for 2 h at 4°C, before serial 40 μm coronal sections were cut on a vibratome from bregma -1.00 to -2.50 mm (Franklin and Paxinos, 2008) and immunolabeled as outlined below. Regions corresponding to the primary somatosensory cortex were analyzed.

### IMMUNOHISTOCHEMISTRY

All immunohistochemical procedures were performed identically. Sections were blocked for 2 h in 10% goat serum and 0.3% Triton-X (both Sigma-Aldrich) by volume in 0.01 M PBS at room temperature, followed by overnight incubation with primary antibodies in blocking solution at 4°C. Human and mouse cortical sections were co-immunolabeled with rabbit anti-CR (1:2000, Millipore) and a cocktail of mouse SMI32 (de-phosphorylated NF) and SMI312 (phosphorylated NF; both at 1:2000, Covance) to visualize CR^+^ and NF^+^ subpopulations of interneurons and principal neurons, respectively. Sections were then counter-stained with 0.0125% thioflavin-S (Sigma-Aldrich) and 40% ethanol in 0.01 M PBS, to visualize fibrillar Aβ plaques. Species-appropriate Alexa 488 and 594 conjugated secondary antibodies (1:500, Molecular Probes) were applied for 2 h at room temperature, followed by extensive washes and mounting with PermaFluor mounting medium (Thermo Fisher Scientific).

### ANALYSIS OF NEURITE SUSCEPTIBILITY

**FIGURE 1:**
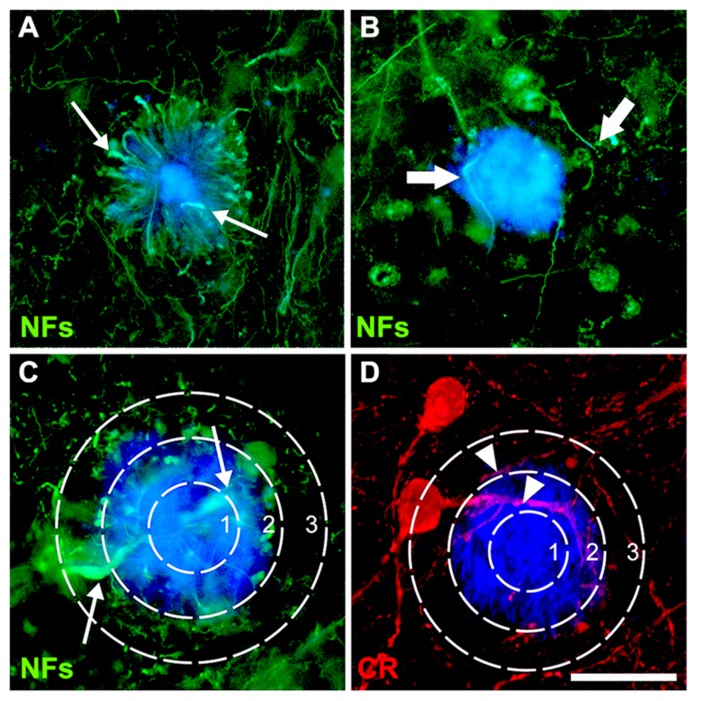
**Double-immunofluorescence for NFs (SMI312 and SMI32) and thioflavin-S (A–C) or calretinin and thioflavin-S (D) with a superimposed template showing the plaque-core, the plaque-edge, and the periphery.** Small arrows **(A,C)** denote characteristic swellings in dystrophic neurites, large arrows **(B)** highlight increased neurite curvature and tortuosity, while arrowheads **(D)** show normal-appearing neurites. Scale bar = 20 μm.

For both AD and mouse cortex, three sections per individual were used, in each of which 10 plaques were chosen (30 plaques per case/animal) to assess their effects on CR^+^ and NF^-^ neurites. Plaques were chosen at random in layers 2–4, which contain the majority of CR^+^ interneuron perikarya and processes in humans (Fonseca and Soriano, 1995; del Rio and DeFelipe, 1997) and mice (Park et al., 2002), as well as the highest plaque load (Braak and Braak, 1991). Using previously described criteria for pathological cytoskeletal alterations, labeled neurites with focal increases in diameter, such as swellings, highly undulating trajectories or with a change in course greater than 90° from the original trajectory, were counted as “dystrophic” (Vickers et al., 1996; Dickson et al., 1999; Knowles et al., 1999; Le et al., 2001; **Figures 1A,B**). Labeled neurites that did not meet any of the dystrophic criteria were considered normal. For each plaque, the number of CR^+^ and NF^+^ neurites traversing the core, edge, and periphery (defined as 50, 100, and 150% of plaque diameter, respectively; **Figures 1C,D**) was determined to assess whether these zones differed in neuropil damage. More than 3000 intact and DNs per case type were counted. The average plaque area for human AD (1290 ± 101 μm^2^) or transgenic mice (1070 ± 67 μm^2^) was used to define plaque-equivalent areas placed randomly in images of control tissue using the grid function in ImageJ, to estimate normal neurite density in these regions. The number of neurites per plaque was normalized to the mean plaque diameter for each case, to allow comparisons of proportions between zones. The mean number of dystrophic and normal neurites in each plaque zone (core, edge, or periphery) was expressed as a percentage of the mean control neurite number. Neurite density loss in AD and transgenic tissue relative to control values was calculated as a percentage: 100 × [1 - (total normal and DNs)/(total neurites in plaque-equivalent control areas)].

To quantify plaque load, five randomly selected sections of inferior temporal gyrus (ITG) neocortex from pia to the white matter were collected for each end-stage AD and preclinical AD case, and an area approximately 1200 μm wide was analyzed in each, as previously described (Woodhouse et al., 2009a). The percentage area that was stained by thioflavin-S was then calculated in ImageJ.

### IMAGE ACQUISITION

Images were captured using a Leica DM LB2 epifluorescence microscope with a cooled CCD Magnafire (Optronics) camera or on a Zeiss LSM510 confocal microscope equipped with Zen software and Ar 488, HeNe 543 lasers. All image analysis was performed using NIH ImageJ (version 1.45p) software.

### STATISTICAL ANALYSIS

Statistical analyses for comparisons of group means was conducted by one-way analysis of variance (ANOVA) followed by Dunnett’s *post hoc* tests or unpaired *t*-tests as appropriate using GraphPad Prism software (version 5.0b) with *p* < 0.05 (CI 95%) considered significant. In some cases, a Pearson product–moment correlation was computed to assess the relationship between DN numbers and mean plaque size. Mean values were reported ± standard error of the mean (SEM).

## RESULTS

### CALRETININ-IMMUNOPOSITIVE INTERNEURONS ASSOCIATED WITH Aβ PLAQUES ARE MORE RESISTANT TO NEURITIC PATHOLOGY THAN NEUROFILAMENT-IMMUNOREACTIVE NEURONS

A cocktail comprising SMI32 and SMI312 antibodies was used to label the NF^+^ subpopulation of neurites, which we assume are chiefly derived from pyramidal cells of cortical layers 2–6 (Van der Gucht et al., 2007; Paulussen et al., 2011). Although neurites from a subset of basket cells, or projecting fibers from subcortical and subthalamic regions, would also be NF^+^ (Hof and Morrison, 1990; Hof et al., 1990), their fewer numbers and less extensive neurite fields indicates that pyramidal neurites make up the majority of NF^+^ processes examined in cortical layers 2–4 (Hof et al., 1995).

**FIGURE 2:**
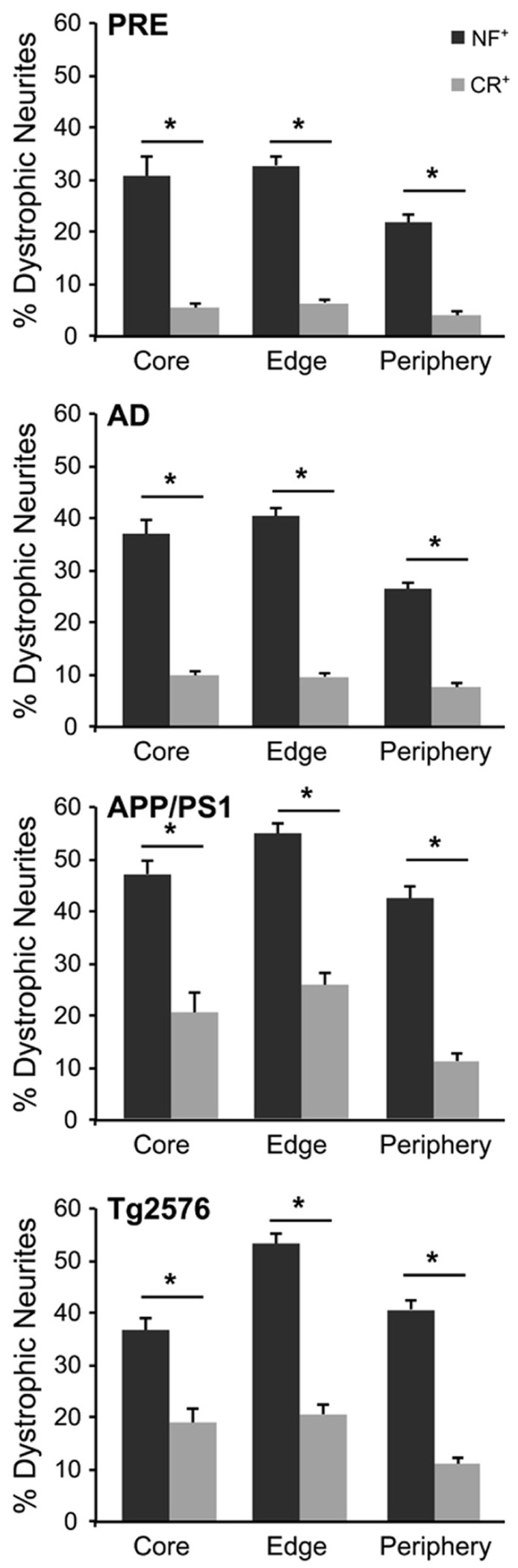
**Graphs illustrating the percentage of NF- (gray bars) and CR- (white bars) immunolabeled dystrophic neurites associated with fibrillar Aβ plaques at the three zones in AD, preclinical AD, APP/PS1, and Tg2576 transgenic mice, respectively (±SEM).** There are significantly more dystrophic NF^+^ neurites traversing the Aβ plaque core, edge, and periphery than CR^+^ dystrophic neurites within all case types (*p < 0.05, *t*-test).

**FIGURE 3:**
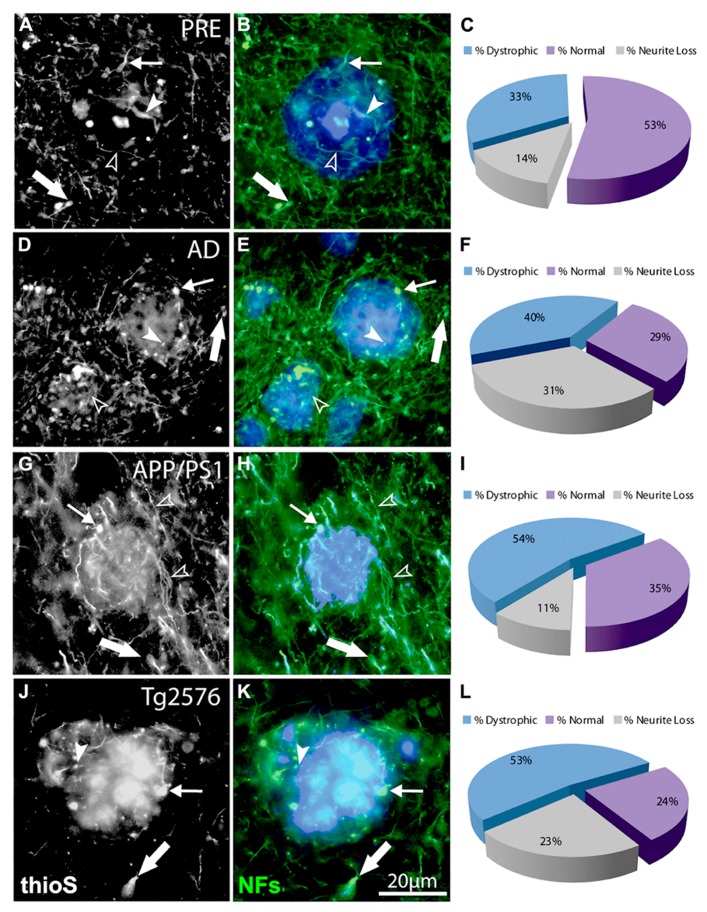
**Double-immunofluorescence labeling of representative thioflavin-S plaques and NF^+^ neurites from preclinical AD (A,B), end-stage AD (D,E), APP/PS1 (G,H), and Tg2576 (J,K) transgenic mice shows classical bulb-like swellings containing neurofilaments indicated by filled arrowheads in the plaque-core, small arrows at the plaque edge, and large arrows in the periphery.** Normal-appearing neurites in association with thioflavin-S labeled fibrillar plaques are indicated by unfilled arrowheads. A large proportion of NF^+^ neurites associated with Aβ plaques are dystrophic (blue fraction: **C,F,I,L**) and there is extensive neruite loss in end-stage AD and Tg2576 mice (gray fraction: **F,L**).

**FIGURE 4:**
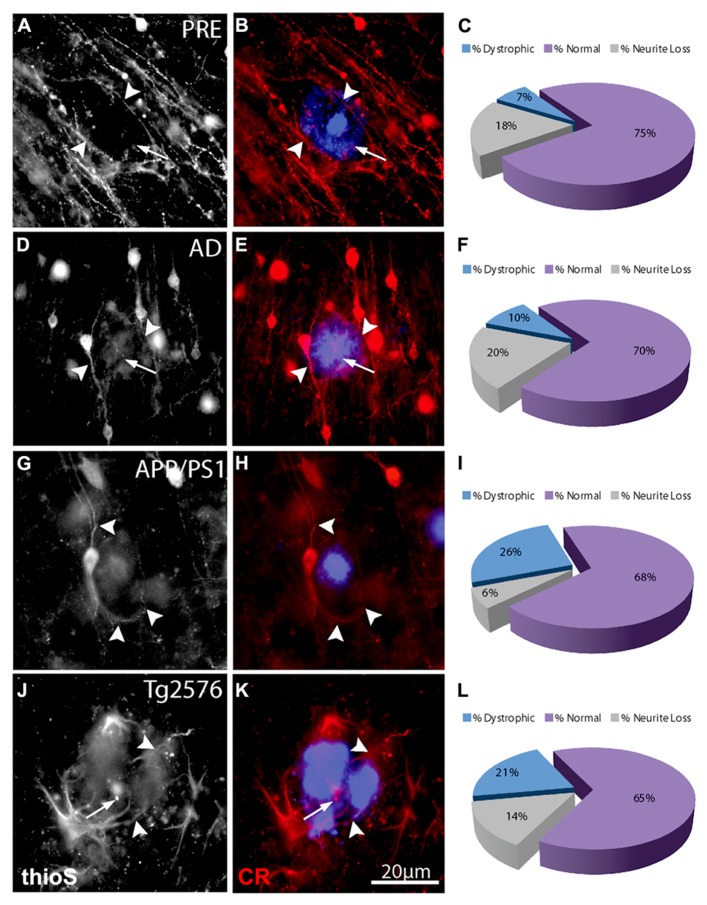
**Double-immunofluorescence labeling of representative thioflavin-S plaques and CR^+^ neurites from preclinical AD (A,B), end-stage AD (D,E), APP/PS1 (G,H), and Tg2576 (J,K) transgenic mice shows a relative paucity of swellings containing calretinin (arrows).** Filled arrowheads indicate normal-appearing CR neurites in association with thioflavin-S labeled fibrillar plaques. In most cases, these neurites appear to elaborate around plaques. A large proportion of CR-ir neurites associated with Aβ plaques are normal (purple fraction: **C,F,I,L**) in all case types and there are comparatively fewer dystrophic neurites (blue fraction: **C,F,I,L**).

The number of NF^+^ and CR^+^ neurites scored as “dystrophic” or “normal” were analyzed at 50, 100, and 150% of Aβ plaque diameters (core, edge, and periphery, respectively) and mean values were expressed as percentages of mean human and mouse control values. For all case types analyzed, the percentage of dystrophic NF^+^ neurites was significantly higher than CR^+^ neurites (**Figure 2**, *p* < 0.01, *t*-test). Of the three zones examined, NF^+^ DNs were most common at the plaque edge in APP/PS1 mice (54.6 ± 2.2%) followed by Tg2576 mice (53.2 ± 2.0%), end-stage AD (40.3 ± 1.7%), and preclinical AD cases (33.4 ± 1.9%; mean ± SEM). Similarly, CR^+^ DNs were most prevalent at the plaque edge in APP/PS1 (25.6 ± 2.5%), Tg2576 (20.5 ± 1.7%), end-stage (9.6 ± 0.8%), and preclinical (6.7 ± 0.7%) AD cases. The greatest difference between NF^+^ and CR^+^ neurite responses in all tissue types was in the plaque periphery, where the proportion of DNs was, on average, four times greater for NF^+^ neurites compared to CR^+^ (**Figures 2–4**). Representative immunolabeled sections demonstrate much greater dystrophy in NF^+^ neurites (**Figure 3**) than in CR^+^ neurites (**Figure 4**).

**FIGURE 5:**
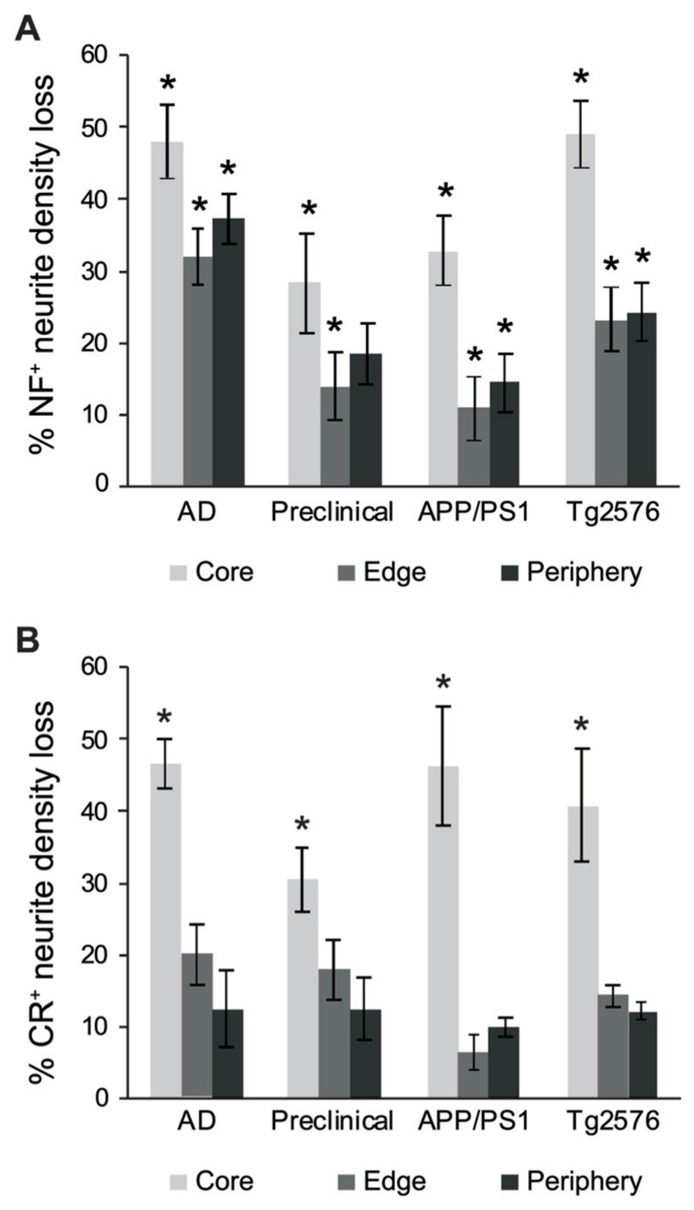
**Graphs showing the percentage neurite density loss for NF^+^ (A) and CR^+^ (B) neurites.** There is a significant NF^+^ neurite density loss for the different case types across all three regions (**A**, *p < 0.01, ANOVA with Dunnett’s *post hoc* test), whereas CR^+^ neurite density loss for the different case types was significant only in the plaque-core (**B**, *p < 0.01). Error bars denote standard error of the mean.

We also counted normal neurites around Aβ fibrillar deposits and in control tissue, to estimate absolute neurite loss. Both NF^+^ (**Figure 5A**) and CR^+^ (**Figure 5B**) neurites exhibited comparable pronounced neurite density loss in the plaque core in end-stage AD, preclinical AD, APP/PS1, and Tg2576 cases (*p* < 0.01, one-way ANOVA, Dunnett’s *post hoc* tests). However, no significant loss of CR^+^ neurites occurred at the plaque edge or periphery (*p* > 0.05 in all cases), whereas NF^+^ neurites showed significant losses in all zones excepting plaque periphery in preclinical AD. Furthermore, in all four case types, the proportion of normal CR^+^ neurites at the plaque edge (**Figures 4C,F,I,L**) was significantly higher than for NF^+^ neurites (**Figures 3C,F,I,L**; *p* < 0.01, *t*-test). Interestingly, many normal CR^+^ neurites in the periphery had elaborated processes around Aβ plaques, instead of traversing the plaque or stopping at the plaque border as NF^+^ neurites often did (arrowheads, **Figure 4**).

### THE NUMBER OF NF-ir DNs IS DEPENDENT ON Aβ PLAQUE SIZE

**FIGURE 6:**
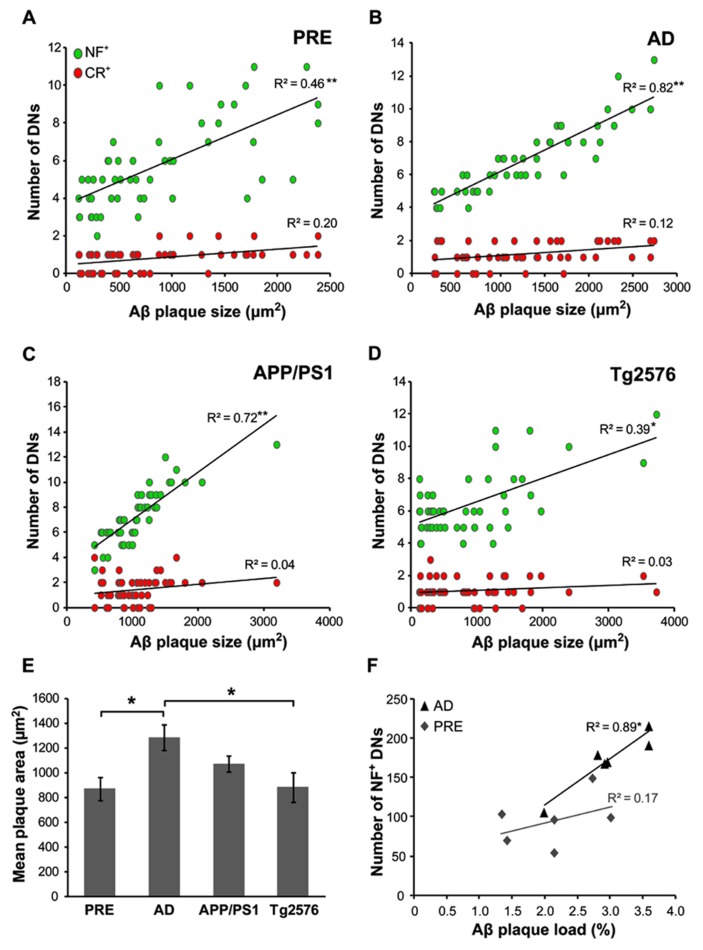
The number of NF^+^, but not CR^+^, dystrophic neurites (DN) was significantly related to Aβ plaque size in both preclinical (A, **p < 0.001, *t*-test) and end-stage AD (B, **p < 0.001), as well as in APP/PS1 (C, **p < 0.001) andTg2576 (D, *p < 0.01, *t*-test) transgenic mice. End-stage AD cases had the largest mean Aβ plaque size (**E**, *p < 0.05, *t*-test). **(F)** The total number of NF^+^ DNs per case correlated significantly with total Aβ plaque load in end-stage AD cases but not with preclinical cases (*n* = 6 each; *p < 0.01, *t*-test). Error bars denote standard error of the mean.

The mean Aβ plaque size was determined in all case types, with the largest plaques occurring in end-stage AD (1290 ± 101 μm^2^) and the smallest in preclinical AD (870 ± 93 μm^2^). In human cases, the mean number of dystrophic NF^+^ neurites correlated with Aβ plaque size in both preclinical (**Figure 6A**, *r* = 0.46, *p* < 0.001, *t*-test) and end-stage AD (**Figure 6B**, *r* = 0.82, *p* < 0.001, *t*-test) cases. There was a similar pattern in the transgenic mice, with higher numbers of NF^+^ DNs associated with larger plaques in APP/PS1 (**Figure 6C**, *r* = 0.72, *p* < 0.001, *t*-test; mean plaque size = 1072 ± 67 μm^2^) and Tg2576 (**Figure 6D**, *r* = 0.39, *p* < 0.01, *t*-test; mean plaque size = 882 ± 118 μm^2^) animals. In contrast, CR^+^ DN counts did not correlate significantly with plaque size in any of the groups (*p* > 0.05 for all). Furthermore, in human end-stage cases, the total number of NF^+^ DNs per case correlated with thioflavin-S plaque load (**Figure 6F**, *r* = 0.89, *p* < 0.05), but not in preclinical AD cases (*r* = 0.17, *p* = 0.12).

## DISCUSSION

Human AD is characterized by the accumulation of extracellular Aβ plaques and intracellular NFTs comprised chiefly of hyperphosphorylated tau (Braak and Braak, 1991; Braak et al., 2011). Interestingly, NF^+^ neurons may be especially vulnerable to developing extensive NFT pathology (Bussiere et al., 2003; Thangavel et al., 2009), as they accumulate large amounts of hyperphosphorylated NFs in their perikarya and axons and eventually degenerate (Morrison et al., 1987; Vickers et al., 1992, 2009; Masliah et al., 1993; Mann, 1996; Su et al., 1996; Dickson et al., 1999; Woodhouse et al., 2009b). However, overt cell death occurs mostly in end-stage AD, and is absent in many transgenic AD models, suggesting that cognitive symptoms which manifest years earlier are due to neuronal dysfunction rather than loss; synaptic and axonal changes may be the earliest functional disruption. Thus, we investigated if NF^+^ neurons were more susceptible to DN formation at early and end-stages of AD, as well as in APP/PS1 and Tg2576 transgenic mice.

Studies in transgenic AD models have previously shown losses in dendritic spine density, increases in neurite curvature, sprouting, and varicosities in pyramidal neuron dendrites (Knowles et al., 1999; Le et al., 2001; Adlard and Vickers, 2002; Spires et al., 2005), as well as axonal disruption (Su et al., 1998; Adalbert et al., 2009; Mitew et al., 2010) associated with Aβ plaques. Likewise, in the current study, we observed a progression of dystrophic changes in NF^+^ neurites from early to end-stage human AD, paralleled in both APP/PS1 and Tg2576 transgenic mice lines. There was an increase in the proportion of dystrophic NF^+^ neurites across all three case types, most prominently at the plaque periphery, then the surrounding neuropil and the plaque core, respectively (**Figure 2**). Concomitantly, the total neurite number relative to age-matched controls decreased markedly, due to depletion in the plaque core and edge (**Figure 5**). This agrees with previous work showing reduced axonal density and total length in AD plaque-associated neuropil (DeWitt and Silver, 1996). The most severe changes occurred in end-stage AD, followed by the Tg2576 and APP/PS1 mouse models, and the least severe in preclinical human AD. The finding that Aβ plaques damage neurites some distance away (i.e., plaque periphery) also concurs with previous studies (Knowles et al., 1999; Le et al., 2001); retrograde swellings in axons have been reported several hundred microns from plaques *in vivo *(Vickers et al., 1996; Adalbert et al., 2009). This implies that dystrophic changes propagate along neurites, or that plaques possess a “halo” of toxicity that extends more distally (Koffie et al., 2009).

In contrast, CR^+^ neurites were less dystrophic in all three plaque zones examined, with significantly more normal-appearing CR^+^ neurites at the edge and in the periphery, but fewer total neurites (normal + dystrophic) in plaque cores. Unlike NF^+^ neurites, which tended to traverse plaque cores, often showing signs of dystrophy, CR^+^ neurites appeared to either terminate at the plaque periphery, or much more commonly, to elaborate around it (**Figure 4**). Furthermore, unlike NF^+^ DNs, counts of CR^+^ DNs did not correlate with plaque size (**Figure 6**). In addition to their potential focal toxicity, larger plaques may physically distort the surrounding neuropil and cause structural alterations to neurites like increased curvature (Knowles et al., 1999; Woodhouse et al., 2005; Meyer-Luehmann et al., 2008). Therefore, CR^+^ neurites may be more resistant to physical damage, or may have a higher capacity for structural remodeling and regeneration. We have recently shown that they exhibit greater structural plasticity than GFP^+^ pyramidal neurons near focal neocortical injury, rearranging their dendritic processes away from the injury site toward unaffected neuropil (Blizzard et al., 2011). Likewise, *in vivo* imaging of adult murine cortex shows that unlike pyramidal neurons, inhibitory neurons frequently extend new dendrites and remodel existing branches (Lee et al., 2008). The results of the present study suggest that fewer CR^+^ neurites traverse plaque cores, while many elaborate processes around plaques (**Figure 4**), potentially indicating dynamic restructuring away from these localized sites of neuropil injury, as previously described (Blizzard et al., 2011).

It is not clear why NF^+^ neurons are selectively vulnerable to AD pathology. One possibility is that elevated Aβ levels interfere with normal NF regulation and transport. Indeed, increased levels of phosphorylation, either at endogenous or at novel NF phosphorylation sites (Liao et al., 2004), are a relatively early event in the pathogenesis of human AD (Wang et al., 2001; Rudrabhatla et al., 2010) and transgenic models (Vickers et al., 1994; Yang et al., 2009). Such aberrant hyperphosphorylation of NFs in AD is likely mediated by abnormal hyperactivity of kinases such as Erk1 and 2 (Veeranna et al., 2004), Cdk5 (Shea et al., 2004), and GSK3β (Chen et al., 2005), or the decreased activity of phosphatases such as PP-2A and 1 (Rudrabhatla et al., 2009). As phosphorylation mediates many aspects of NF function including axonal transport of NFs and radial growth of axons, dysregulation of this system could critically impact cytoskeletal integrity in AD (Shea and Chan, 2008). Higher NF expression and phosphorylation is also associated with maturation of neurons resulting in larger axonal caliber and myelination, effectively increasing structural stability at the expense of plasticity. Therefore, a combination of lower capacity for structural remodeling and a perturbed phosphorylation balance could prime NF^+^ neurites for DN formation and cytoskeletal dysfunction in AD.

In conclusion, we report that CR^+^ interneurons are less susceptible than NF^+^ neurons to neurite damage mediated by Aβ plaques, in both human AD cases and transgenic mouse models. It is likely that a combination of factors, such as a higher capacity for structural remodeling and regeneration following focal injury, contribute to this relative resistance. This study contributes to previous work showing that NFs may predispose the subpopulations of neurons that express them to structural damage in AD (Hof and Morrison, 1990; Hof et al., 1990, 1993; Vickers et al., 1996, 2009; Sampson et al., 1997).

## Conflict of Interest Statement

The authors declare that the research was conducted in the absence of any commercial or financial relationships that could be construed as a potential conflict of interest.
